# 638. Using NHSN Annual Self-Reported Dialysis Data to Understand Infection Prevention and Control Practices and their Association with Bloodstream Infections in Dialysis Facilities

**DOI:** 10.1093/ofid/ofae631.203

**Published:** 2025-01-29

**Authors:** Rabia R Syed, Lacey Pavlovsky, Kate Tyner, Dan K German, Christina Cashatt, Daniel M Brailita, Muhammad Salman Ashraf, Juan M Teran Plasencia

**Affiliations:** Nebraska Department of Health and Human Services, Lomita, CA; Nebraska Department of Health and Human Services, Lomita, CA; Nebraska Medicine, Omaha, NE; Nebraska Medicine, Omaha, NE; Nebraska ICAP, Papillion, Nebraska; Division of Infectious Diseases, University of Nebraska Medical Center, Omaha Nebraska;Nebraska Infection Control Assessment and Promotion Program, Nebraska Medicine, Omaha Nebraska, Omaha, Nebraska; University of Nebraska Medical Center; University of Nebraska Medical Center/Division of Infectious Diseases, Omaha, Nebraska

## Abstract

**Background:**

Hemodialysis (HD) patients are at increased risk of bloodstream infection (BSI). Although BSI events are surveilled by the National Healthcare Safety Network (NHSN), studies correlating dialysis facilities’ self-reported infection control (IC) practices with BSI events are lacking.

**Figure 1.**
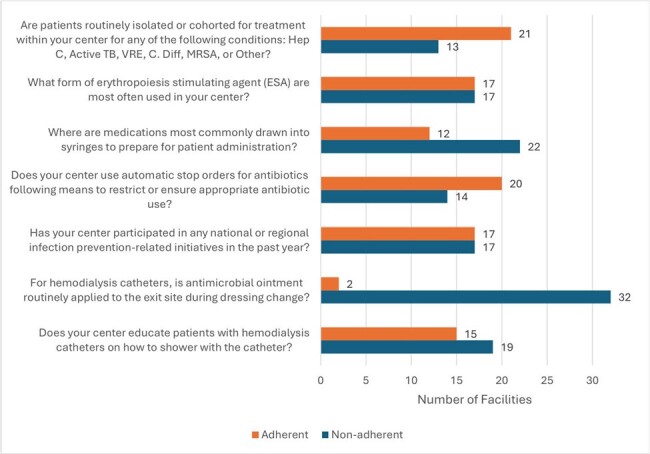
Seven Areas of Least Compliance by Facilities

A total of 34 facilities were included. Best practices were selected based on adherence by less than 2/3 of dialysis centers.

**Methods:**

We used 2022 NHSN Annual Outpatient Dialysis Center Practices Survey data for Nebraska facilities to identify 44 questions pertaining to IC practices. These questions were included into a scoring system and dichotomized to adherent (1) or non-adherent (0), leading up to a “best practice (BP) score” for each facility. Questions related to attending IC training initiatives were coded as yes (1) or no (0). Self-reported adherence to CDC Dialysis Safety Core Interventions (DSCI) were coded as following all (1) or some/none (0). We extracted BSI standardized infection ratios (SIR) from NHSN Dialysis Events Surveillance data. Descriptive statistics was used to evaluate facility characteristics and scores. Multivariate regression, using a Poisson model, generated rate ratios (RR) to understand association of trainings/guidelines with BP scores and BSI SIR. Pearson correlation was used to assess association between BSI SIR and BP scores.

**Figure 2.**
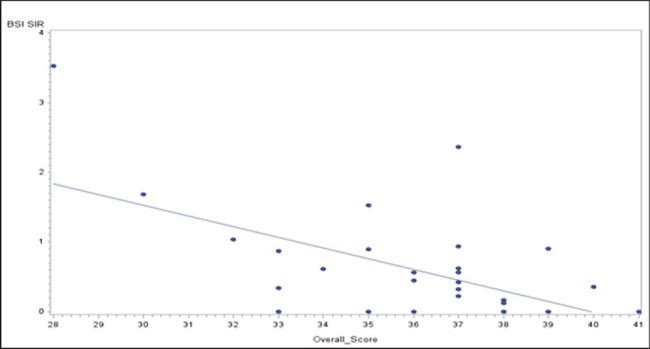
BSI SIR and Overall Facility Performance Score Distributions

**Results:**

A total of 34 facilities yielded a mean BP score of 36/44 (82%). The top 3 least reported BP strategies included: ointment application to HD catheter during dressing change (adhered by 2 of 34), medications drawn into syringes outside the patient room (adhered by 12 of 34), and patient education on showering with HD catheters (adhered by 15 of 34). Overall, facilities following all DSCI were associated with higher BP scores (RR: 1.11, 95% CI: 1.06, 1.16) and lower BSI SIR (RR: 0.33, 95% CI: 0.13, 0.83) compared to those who followed some or none. Similarly, facilities participating in infection prevention initiatives were associated with lower BSI SIR (RR: 0.39, 95% CI: 0.16, 0.96) compared to those who did not. An inverse association was found between BSI SIR and BP score (p=0.001).

**Conclusion:**

Adherence to DSCI and participation in IC initiatives likely help HD facilities in preventing BSI events and need to be further explored. NHSN annual outpatient dialysis data provides important information about areas where further improvement is needed.

**Disclosures:**

**Muhammad Salman Ashraf, MBBS**, Merck & Co. Inc: Grant/Research Support

